# Jaw osteosarcoma models in mice: first description

**DOI:** 10.1186/s12967-019-1807-5

**Published:** 2019-02-27

**Authors:** Hélios Bertin, Romain Guilho, Régis Brion, Jérôme Amiaud, Séverine Battaglia, Anne Moreau, Anne Brouchet-Gomez, Julie Longis, Benoit Piot, Dominique Heymann, Pierre Corre, Françoise Rédini

**Affiliations:** 1 Laboratoire des sarcomes osseux et remodelage des tissus calcifiés (Phy.OS), UMR 1238, Faculté de médecine, 1 rue Gaston Veil, 44035 Nantes Cedex, France; 2 Service de chirurgie Maxillo-faciale et stomatologie, CHU de Nantes, 1 place Alexis Ricordeau, 44093 Nantes Cedex 1, France; 3 Faculty of Population Health Sciences, UCL Institute of Child Health, 30 Guilford Street, London, England WC1N 1EH UK; 4 Service d’anatomie et cytologie pathologique, CHU de Nantes, 1 place Alexis Ricordeau, 44093 Nantes Cedex 1, France; 5 Service d’anatomie et cytologie pathologique, Institut Universitaire du Cancer Toulouse Oncopôle, 1 avenue Irène Joliot-Curie, 31059 Toulouse Cedex 9, France; 6 Centre de ressources biologiques – Cancer, Institut Universitaire du Cancer Toulouse Oncopôle, 1 avenue Irène Joliot-Curie, 31059 Toulouse Cedex 9, France; 7 Laboratoire Hétérogénéité Tumorale et Médecine de Précision, Institut de Cancérologie de l’Ouest, Boulevard Jacques Monod, 44805 Saint Herblain, France; 8 Service d’Histologie-Embryologie, Faculté de médecine de Nantes, 1 Rue Gaston Veil, 44035 Nantes, France; 9 Laboratoire d’Ingénierie Ostéo-Articulaire et Dentaire (LIOAD), Faculté de Chirurgie Dentaire, 1 Place Alexis Ricordeau, 44042 Nantes, France

**Keywords:** Sarcoma, Mandible, Models, Environment, Animal experimentation, Bone resorption

## Abstract

**Background:**

Osteosarcoma (OS) is the most common cancer of bone. Jaw osteosarcoma (JOS) is rare and it differs from other OS in terms of the time of occurrence (two decades later) and better survival. The aim of our work was to develop and characterize specific mouse models of JOS.

**Methods:**

Syngenic and xenogenic models of JOS were developed in mice using mouse (MOS-J) and human (HOS1544) osteosarcoma cell lines, respectively. An orthotopic patient-derived xenograft model (PDX) was also developed from a mandibular biopsy. These models were characterized at the histological and micro-CT imaging levels, as well as in terms of tumor growth and metastatic spread.

**Results:**

Homogeneous tumor growth was observed in both the HOS1544 and the MOS-J JOS models by injection of 0.25 × 10^6^ and 0.50 × 10^6^ tumor cells, respectively, at perimandibular sites. Histological characterization of the tumors revealed features consistent with high grade conventional osteosarcoma, and the micro-CT analysis revealed both osteogenic and osteolytic lesions. Early metastasis was encountered at day 14 in the xenogenic model, while there were no metastatic lesions in the syngenic model and in the PDX models.

**Conclusion:**

We describe the first animal model of JOS and its potential use for therapeutic applications. This model needs to be compared with the usual long-bone osteosarcoma models to investigate potential differences in the bone microenvironment.

**Electronic supplementary material:**

The online version of this article (10.1186/s12967-019-1807-5) contains supplementary material, which is available to authorized users.

## Background

Osteosarcoma (OS) is the most common cancer of bone [1, 2]. Jaw osteosarcoma (JOS) is a rare condition, representing only 5 to 10 percent of all osteosarcomas [3–5]. It differs from long-bone osteosarcomas (LBOS) as it typically occurs two decades later [5, 6], has a lower metastatic potential [4], and has better patient survival rates [6, 7]. Metastases remain the major cause of death [8]. The treatment of JOS is based on the treatment of LBOS, and it comprises neoadjuvant chemotherapy followed by surgical resection and adjuvant chemotherapy [9, 10]. The surgical procedure remains complicated with facial locations because it is difficult to obtain free surgical margins, thus leading to functional and aesthetic impairments. Radical surgery with wide clear margins is the main prognostic factor of the disease [6, 11]. The rarity of the disease makes it difficult to carry out early-phase clinical trials. There is, therefore, a need for appropriate animal models that recapitulate the complexity and the heterogeneity of this malignancy [12].

OS is characterized by a lack of recurrent translocations and a complex karyotype [13]. The tumor suppressor genes p53 and Rb are frequently altered and they appear to be involved in initiation of the disease [14–16]. It has also been well established that the bone microenvironment plays a major role in the development and progression of osteosarcomas [17, 18]. The in vivo interactions between tumor cells and the host are still largely unknown [2]. The generation of specific animal models that mimic the human disease would allow for a better understanding of the mechanisms involved in tumorigenesis and to test new therapeutic agents.

Several OS mouse models have been developed in the long bones [19]. But to date, no specific animal model of JOS has been carried out, and the biological behaviour of these sarcomas appears to differ from LBOS [20]. The aim of our work was to develop and characterize specific mouse models of JOS.

## Methods

### Animal housing and handling

Four-week-old female mice (Elevages Janvier, France) were housed in groups of four under pathogen-free conditions at the Experimental Therapy Unit (Medical School, Nantes, France) in accordance with French institutional guidelines (CEEA.PdL.06, authorization no 8405 and 8449). Mice were given access to food and water ad libitum. This report adheres to the EU directive 2010/63/EU and the ARRIVE Guidelines for reporting animal research [22], and a completed checklist is included in Additional file 1.

### Transplantation of murine and human osteosarcoma cell lines

Osteosarcoma cell lines (Additional file 2: Table S1) suspended in PBS solution were inoculated under general anaesthesia at a paraosseous site after periosteum scraping (isoflurane-air mixture, 1.5%, 1 L/min). For ethical considerations, mice were euthanized by cervical dislocation when the tumor volume reached 150 mm^3^.

Three murine (MOS-J, POS-1, and K7-M2) and four human (HOS1544, HOS 1547, MG-63, and SaOS-2) LBOS cell lines were used. One million cells were injected at a mandibular site in 7 groups of 3 mice (C57Bl/6, C3H/HeN, and Balb/c for the syngenic models, and in NMRI-nude mice for the xenogenic models). A dose–effect study was carried out: 0.25 × 10^6^, 0.50 × 10^6^, and 1 × 10^6^ MOS-J or HOS1544 cells as a 20 μL suspension in PBS in C57Bl/6 and NMRI-nude mice (n = 4/group).

### Development of a JOS PDX model from a patient

PDX models were developed from the biopsy of a patient exhibiting a JOS. A 1 mm^3^ fragment of the tumor was grafted under the mandibular or the tibial periosseous membrane in two anesthetized SCID mice (xylazine 8%-ketamine 5% in PBS; 100 μL/10 g). Part of the specimen was digested for 2 h at 37 °C in Dulbecco’s modified Eagle’s medium (DMEM) (Biowhittaker, Belgium), 10% collagenase, and 1% Penicillin 100 U/mL—Streptomycin 100 mg/L (Invitrogen, France). After centrifugation, the cells (hereafter referred to as AT2015 cells) were cultured at 37 °C in DMEM/10% FBS, 1% glutamine, and 1% antibiotics. The cells were washed in DMEM and then used for perimandibular (1 × 10^6^; 20 μL) and paratibial injection (2 × 10^6^; 40 μL).

### Tumor growth recordings

Tumor volumes were calculated using the formula (l^2^ × L)/2, where l is the smallest and L the largest perpendicular diameter of the tumor.

### Micro-CT analysis

Mandibles were scanned at necropsy using a SkyScan-1072 X-ray microcomputed tomography system (Bruker, Massachusetts, USA) with the following parameters: 18 μm pixel size, 50 kV, 0.5-mm AI filter, and a rotation step of 0.7 degrees. Three-dimensional (3D) reconstructions and analysis were performed using NRecon, CTVox and CTAn 32-bit software (Skyscan). A region of interest corresponding to the tumor area was defined from the posterior side of the central incisor over a length of 5.4 mm. The cortical (BV, BV/TV) and trabecular (Tb.Th, Tb.Sp and Tb.N) bone parameters were quantitatively compared between tumor mandibles and normal jaw in the HOS1544 model.

### Histological analysis

Jaws were fixed in 10% buffered formaldehyde and then decalcified by electrolysis. After embedding in paraffin, 3-μm-thick sections were stained with haematoxylin–eosin (HE) or with Masson trichrome. For the analysis of lung metastases, 3 μm-thick sections were generated every 300 μm and the tumor foci were quantified using NDPView2 Hamamatsu software (SZK, Japan).

### Statistical analysis

The statistical analysis was performed with GraphPad Prism software for Windows (GraphPad Software, La Jolla, USA). A two-factor ANOVA and the unpaired Mann–Whitney test were used to compare the tumor volumes and the number of metastases, respectively. An unpaired Mann–Whitney test and a Wilcoxon test for paired observations allowed to analyse the bone parameters. Results with P < 0.05 were considered significant.

## Results

### Development of preclinical models of mouse JOS

All of the mice injected with murine osteosarcoma cells developed a mandibular tumor. For the syngenic models, the tumor growth was more homogeneous in the MOS-J model than POS-1 and K7M2, with a mean tumor volume of 46.6 mm^3^ (± 11.5 mm^3^), 10 mm^3^ (± 14.1 mm^3^), and 15.3 mm^3^ (± 11.5 mm^3^), respectively, at day 11 (D11). For the xenogenic models, the most homogeneous growth was observed with HOS1544 cells: mean tumor volume 60 mm^3^ (± 20 mm^3^) at D11 versus 73.3 mm^3^ (± 30.5 mm^3^), 60 mm^3^ (± 34.6 mm^3^), and 33.3 mm^3^ (± 11.5 mm^3^) for the MG-63, HOS1547, and SaOs-2 cells, respectively.

Uniform dose-dependent growth of the mandibular tumor was observed in the HOS1544 model (p = 10^−4^), while the MOS-J model was more heterogeneous. The most comparable growth was obtained with inoculation of 0.25 × 10^6^ HOS1544 cells and 0.5 × 10^6^ MOS-J cells, respectively (Additional file 2: Figure S1).

PDX models developed at both mandibular and tibial sites irrespective of whether a tumor fragment or AT2015 tumor cells were used. A palpable mass was detected at D14 for the tumor graft and D60 for the tumor cell inoculation. The tumor volume reached 100 mm^3^ at D30 and at D75 for the respective models.

### Characterization of the JOS models

#### Histological analysis

**Fig. 1:**
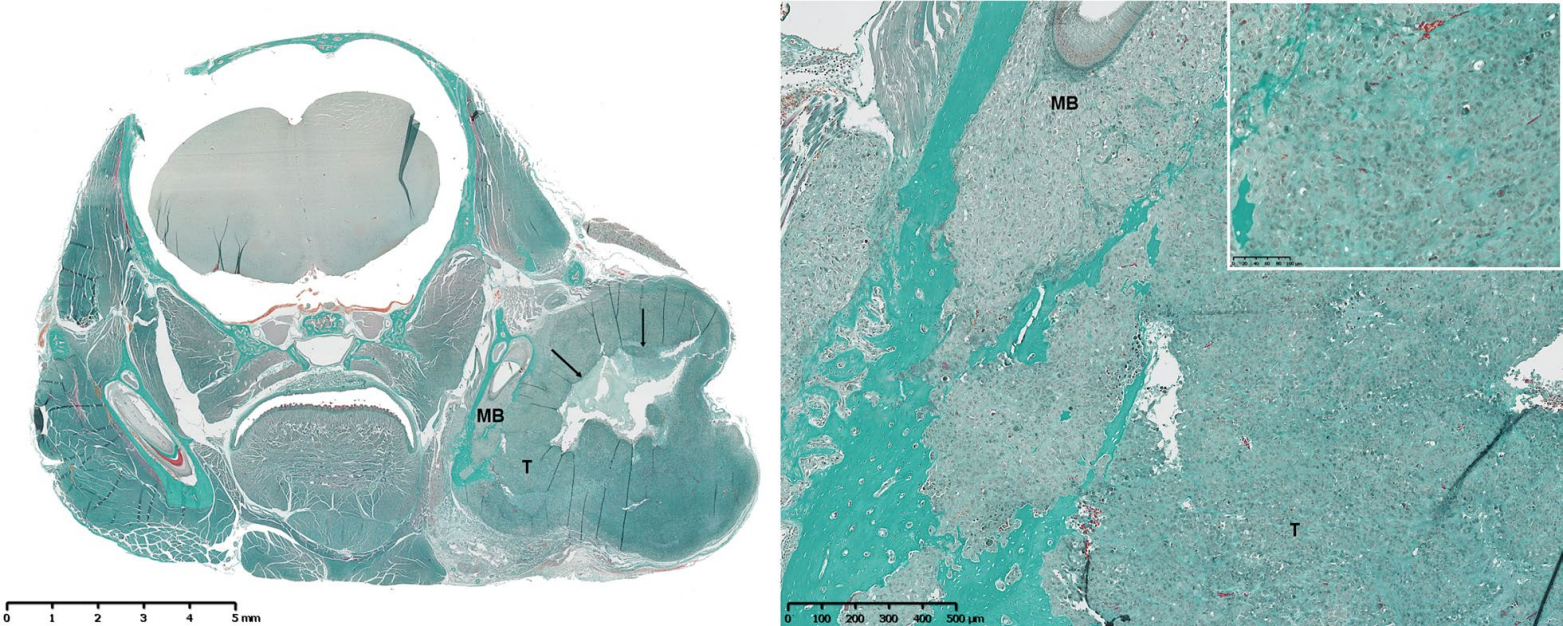
Masson’s trichrome staining of a section of a mandibular osteosarcoma developed in an NMRI-nude mouse (HOS1544 model) in a frontal view (left, original magnification (OM)). Note the large tumor (T) with local invasion of the cortical and medullary mandibular bone (MB), and substantial central tumor necrosis (black arrows) (right, OM × 5). The osteoblastic tumor cells present with several mitoses and produce intercellular osteoïd substance (window, OM x 20)

**Fig. 2:**
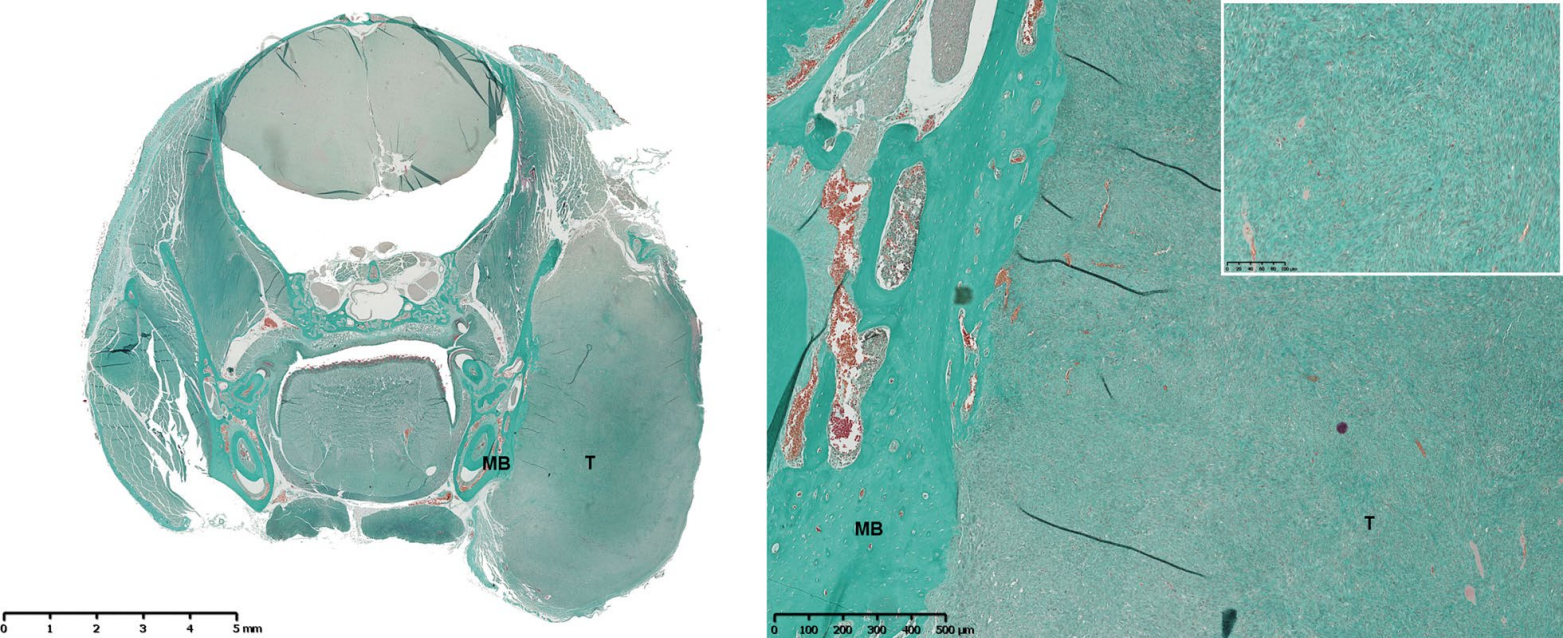
Masson’s trichrome staining of a section of a mandibular osteosarcoma developed in C57Bl/6 mouse (MOS-J model) in a frontal view (left, original magnification (OM)). Large tumor (T) developed in contact with left mandibular bone (MB) (right, OM × 5). High density of osteoblastic tumor cells secreting osteoïd substance (window, OM × 20)

Histological examination confirmed the diagnosis of high grade conventional osteosarcomas exhibiting multiple osteoblastic tumor cells, with polymorphic nuclei, thin chromatin, and large nucleoli. Areas of intratumoral necrosis were often observed, together with several mitoses irrespective of whether xenogenic (Fig. 1) or syngenic (Fig. 2) models were being studied.

**Fig. 3:**
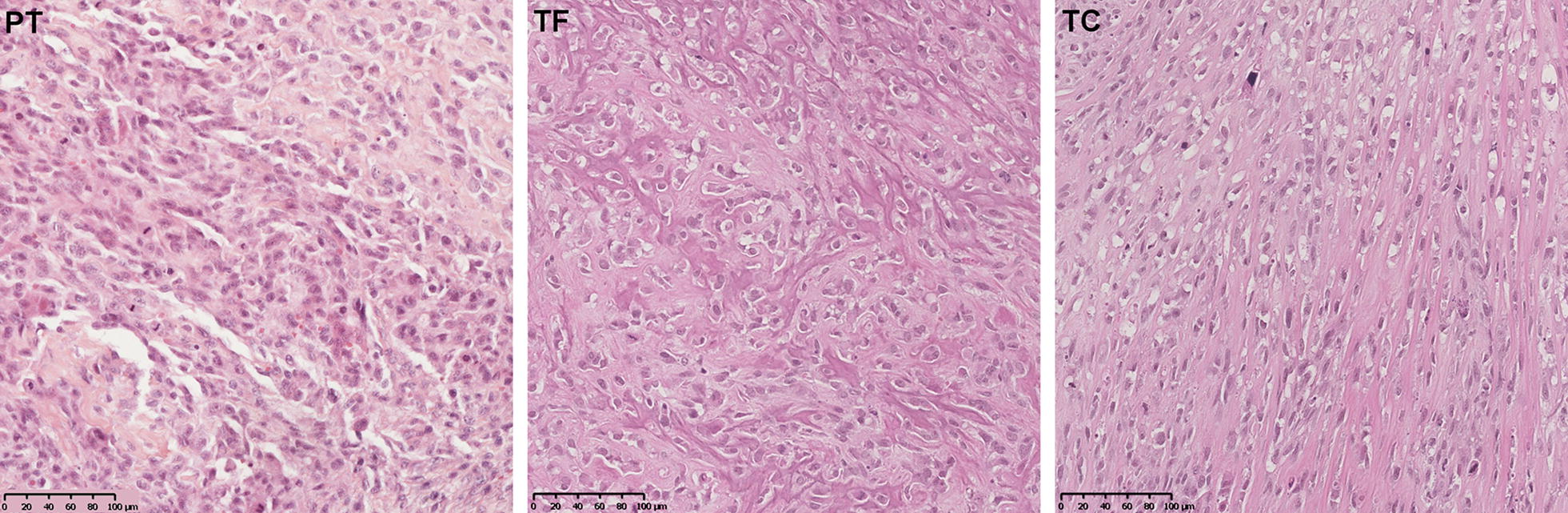
Histological comparison of the osteosarcoma parental tumor (PT) (left) with the PDX models derived from a tumor fragment (TF) (middle) and from the AT2015 tumor cells (TC) (right) (OM × 20). Conventional osteosarcoma features with osteoblastic tumor cells in an osteoid stroma, various nuclear atypia with high mitotic index. The rare areas of chondroblastic differentiation are not showed in this section

The PDX models were compared with the human parental tumor, exhibiting very similar lesions, irrespective of the use of a tumor fragment or tumor cell injection (Fig. 3). This analysis revealed high grade osteosarcoma features with osteoblastic tumor cells that secreted an osteoid substance and that had various nuclear atypia with a high mitotic index and focal areas of chondroblastic differentiation.

#### Micro-CT analysis

**Fig. 4:**
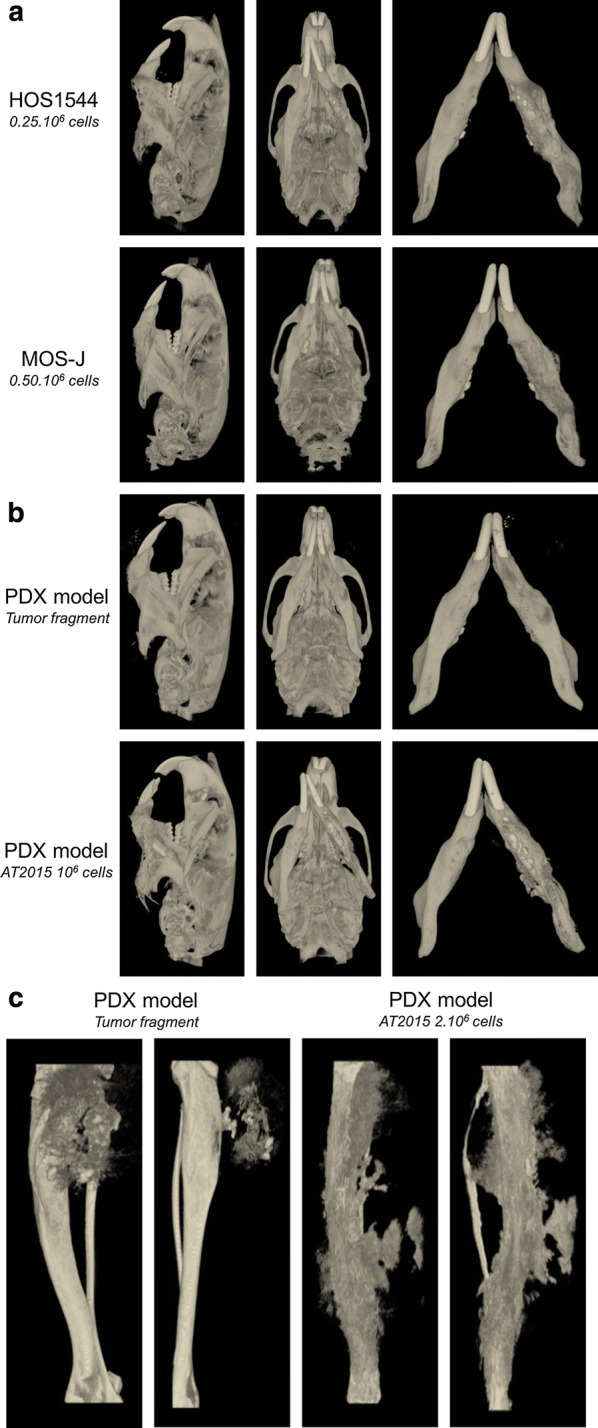
Micro-CT analysis of the xenogenic HOS1544 and syngenic MOS-J JOS models (**a**). Micro-CT characterization of the JOS (**b**) and tibial OS (**c**) PDX models

The bone lesions were comparable between the murine models and the human disease. A high prevalence of mandibular osteolytic lesions was observed in the HOS1544 and the AT2015-induced PDX models. More osteogenic features were noted in the MOS-J model and in the PDX induced with a human tumor fragment (Fig. 4a, b). The quantitative analysis of bone parameters in the HOS1544 model in comparison with contralateral normal mandible in mice showed a trend of tumor induced bone lysis, as revealed by the decrease of BV, BV/TV, Tb.Th, Tb.N and the increase of Tb.Sp but with non-statistical significance (Table 1).

**Table 1:** Cortical (BV, BV/TV) and trabecular (Tb.Th, Tb.Sp, Tb.N) bone parameters measured in tumor and contralateral (normal) mandibles

	Tumor jaw (n = 8)	Normal jaw (n = 8)
Bone volume (BV), mean ± SD (mm3)	12.64 ± 1.21	13.09 ± 0.27
BV/Tumor volume (BV/TV)	61.17 ± 7.09	66.57 ± 0.96
Trabecular thickness (Tb.Th), mean ± SD (mm)	0.29 ± 0.02	0.30 ± 0.01
Trabecular separation (Tb.Sp), mean ± SD (mm)	0.27 ± 0.04	0.24 ± 0.01
Trabecular number (Tb.N), mean ± SD	2.09 ± 0.17	2.22 ± 0.03

*N* number of animals,* SD* standard deviation

Large osteogenic lesions with a high level of periosteal reaction were observed in the PDX models induced in long bones, particularly those induced with AT2015 tumor cells (Fig. 4c).

#### Tumor growth and metastatic spread in lungs

**Fig. 5:**
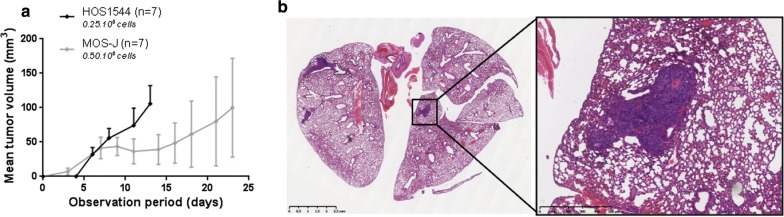
Comparative analysis of the mean mandibular tumor volume over time in the HOS1544 and the MOS-J JOS models (**a**). Haematoxylin–eosin staining of a section of the lungs of an NMRI-nude mouse in a frontal view (OM), showing the presence of three metastases in both lungs, presenting as clusters of cells with large nuclei and a well-vascularized pattern (**b**)

The mandibular tumor grew significantly faster in the xenogenic HOS1544 JOS model compared to the syngenic MOS-J model, with the tumor volume reaching 100 mm^3^ at D14 versus D23, respectively (Fig. 5a). There was substantial metastatic spread in lungs in the HOS1544 JOS model, with a mean number of metastases of 5.6 (0-12) per mouse at D14. This value did not correlate with the mean tumor volume. Most of the lung lesions were macroscopically visible (Fig. 5b). Neither lung metastases could be discerned in the MOS-J JOS model at D23, nor in the PDX models.

## Discussion

As the survival rate for osteosarcoma has not changed over the past 40 years [19, 23, 24], the development of new and more effective therapeutic approaches, as assessed with relevant preclinical models [25], is urgently needed [12].

An ideal animal model of osteosarcoma should recapitulate all of the aspects of the human pathology, from the genetic events to the functional osteoid matrix production by osteosarcoma cells [2], and it should also disseminate spontaneously to the lungs [8]. To date, several OS mice models have been developed in the long bones [19]. They are induced by different approaches; radiation- and chemically induced mouse models refer to DNA damage studies [2, 13], while genetically engineered mouse (GEM) models have been induced by deletion of p53 and Rb in the osteoblast lineage [23, 26], as well as by overexpression of oncogenes in osteoblastic precursors [27]. These models help with gaining a better understanding of the molecular mechanisms involved in tumor initiation [24], but they remain heterogeneous in terms of specificity, incidence, and tumor latency [26]. Tumor cell graft models are routinely used in mice, as they are easy to set-up, affordable, and reproducible [2, 13, 19, 25]. They allow for a better understanding of the mechanisms involved in tumor and metastatic progression [13]. Patient-Derived Xenograft (PDX) models based on grafting of neoplastic cells or tissues obtained from patients in immunodeficient mice [8, 28] appear to better reproduce the tumor microenvironment and allow a wide range of drugs to be tested by means of a personalized approach for patients [29–31]. In regard to our study, the HOS1544 and MOS-J cell lines have been reported to induce a primary tumor after paratibial or intraosseous injection in nude mice and C57Bl/6 mice, respectively, and to allow for spontaneous lung metastasis [19, 32]. Unfortunately, these models do not, however, recapitulate tumor initiation and they do not account for the impact of the bone microenvironment, particularly the immune response that occurs in xenogenic models [2, 13].

We here describe the first JOS model in non-genetically modified mice, induced by inoculation of HOS1544 and MOS-J cells in close contact with the mandible. These models were found to reproduce the same histological and morphometric characteristics of the human disease at the jaw site. Early metastatic spread was observed in the HOS1544 model, as well as fast and homogeneous tumor growth, as previously described for LBOS models and correlated with the high level of aggressiveness of this cell line [33]. However, for ethical considerations, early euthanasia of the animals has to be performed when the tumor volume reaches 150 mm^3^ at the jaw site, while a 1500 mm^3^ tumor volume is acceptable in long bones.

We concomitantly developed orthotopic PDX models of jaw osteosarcoma in SCID mice, which, to our knowledge, represents the first description at a mandibular location. The main advantage of this model is that it preserves the native tumor microenvironment, allowing primary tumor formation and the early stage of metastatic progression to be studied [13, 28]. PDX models are particularly valuable with rare cancers, due to the small number of patients eligible for evaluation of experimental therapies and the possibility to test personalized treatments [30, 31]. Some limitations arise due to the need for a sufficient amount of fresh tissue and the technical ability to achieve engraftment, which is a lengthy process that suffers from a low rate of success and high cost [12, 28, 29, 31]. Although PDX have an identical genomic profile as the original tumor [25], a careful histologic analysis of PDX and their parental tumors is recommended prior to their use in preclinical analyses [12].

OS is a malignant tumor of mesenchymal origin that simultaneously generated osteolytic lesions and mineralized osteoid matrix, thereby resulting in mixed lytic/blastic lesions. The bone-morphometric parameters of our models were investigated by micro-CT in order to analyse the functional behaviour of the tumors. Osteogenic lesions were observed in the MOS-J-induced mice and osteolytic lesions in the HOS1544, POS-1, and K7M2 models, in accordance with those previously reported in the respective paratibial models.

The differences observed in the clinical and biological behaviour between the two sites may be due to different microenvironments, although there have been very few studies to date comparing the tumor microenvironment in LBOS versus JOS [34, 35]. Vascularization could play an important role since immunohistochemical studies have revealed a significantly lower level of VEGF expression in JOS compared to LBOS samples, which may explain a lower metastatic potential of the jaw site [34]. In addition, differences in the periosteal reaction observed in the PDX models, whether the AT2015 tumor cells were injected at tibial or mandibular sites, can be explained by variations in the equilibrium between bone formation and resorption at the tumor site.

Further studies are hence needed to assess the role of the jaw microenvironment in osteosarcoma development by investigation of the immune response to tumor initiation, vascularization, and bone remodelling. Our new JOS models could prove to be a key resource for such studies and for testing of new therapeutic agents.

## Conclusion

We described the first animal model of JOS and its potential use in therapeutic applications. This model needs to be compared with conventional long-bone osteosarcoma models to investigate potential bone microenvironment differences.

## Additional files

**Additional file 1.** The arrive guidelines checklist.

**Additional file 2: Table S1.** Murine and human osteosarcoma cell lines used for the development of syngenic and xenogenic JOS animal models. **Figure S1**. Mean mandibular tumor volume over time, as a function of tumor cell number injected in the xenogenic HOS1544 (A) or the syngenic MOS-J (B) models in NMRI-nude or C57Bl/6 mice respectively.

## References

[1] Lacour B, Guyot-Goubin A, Guissou S, Bellec S, Désandes E, Clavel J (2010). Incidence of childhood cancer in France: National Children Cancer Registries, 2000–2004. Eur J Cancer Prev Off J Eur Cancer Prev Organ ECP.

[2] Ek ETH, Dass CR, Choong PFM (2006). Commonly used mouse models of osteosarcoma. Crit Rev Oncol Hematol.

[3] Ottaviani G, Jaffe N (2009). The epidemiology of osteosarcoma. Cancer Treat Res.

[4] Thariat J, Julieron M, Brouchet A, Italiano A, Schouman T, Marcy P-Y (2012). Osteosarcomas of the mandible: are they different from other tumor sites?. Crit Rev Oncol Hematol.

[5] van den Berg H, Schreuder WH, de Lange J (2013). Osteosarcoma: a comparison of Jaw versus Nonjaw localizations and review of the literature. Sarcoma..

[6] Thariat J, Schouman T, Brouchet A, Sarini J, Miller RC, Reychler H (2013). Osteosarcomas of the mandible: multidisciplinary management of a rare tumor of the young adult a cooperative study of the GSF-GETO, Rare Cancer Network, GETTEC/REFCOR and SFCE. Ann Oncol Off J Eur Soc Med Oncol ESMO.

[7] Nissanka EH, Amaratunge EAPD, Tilakaratne WM (2007). Clinicopathological analysis of osteosarcoma of jaw bones. Oral Dis.

[8] Wagner F, Holzapfel BM, Thibaudeau L, Straub M, Ling M-T, Grifka J (2016). A validated preclinical animal model for primary bone tumor research. J Bone Joint Surg Am.

[9] Luetke A, Meyers PA, Lewis I, Juergens H (2014). Osteosarcoma treatment—where do we stand? A state of the art review. Cancer Treat Rev.

[10] Crenn V, Biteau K, Amiaud J, Dumars C, Guiho R, Vidal L (2017). Bone microenvironment has an influence on the histological response of osteosarcoma to chemotherapy: retrospective analysis and preclinical modeling. Am J Cancer Res.

[11] Granowski-LeCornu M, Chuang S-K, Kaban LB, August M (2011). Osteosarcoma of the jaws: factors influencing prognosis. J Oral Maxillofac Surg Off J Am Assoc Oral Maxillofac Surg.

[12] Lu W, Chao T, Ruiqi C, Juan S, Zhihong L (2018). Patient-derived xenograft models in musculoskeletal malignancies. J Transl Med.

[13] Guijarro MV, Ghivizzani SC, Gibbs CP (2014). Animal models in osteosarcoma. Front Oncol.

[14] Wadayama B, Toguchida J, Shimizu T, Ishizaki K, Sasaki MS, Kotoura Y (1994). Mutation spectrum of the retinoblastoma gene in osteosarcomas. Cancer Res.

[15] Toguchida J, Yamaguchi T, Ritchie B, Beauchamp RL, Dayton SH, Herrera GE (1992). Mutation spectrum of the p53 gene in bone and soft tissue sarcomas. Cancer Res.

[16] Miller CW, Aslo A, Won A, Tan M, Lampkin B, Koeffler HP (1996). Alterations of the p53, Rb and MDM2 genes in osteosarcoma. J Cancer Res Clin Oncol.

[17] Rubio R, Abarrategi A, Garcia-Castro J, Martinez-Cruzado L, Suarez C, Tornin J (2014). Bone environment is essential for osteosarcoma development from transformed mesenchymal stem cells. Stem Cells Dayt Ohio.

[18] Alfranca A, Martinez-Cruzado L, Tornin J, Abarrategi A, Amaral T, de Alava E (2015). Bone microenvironment signals in osteosarcoma development. Cell Mol Life Sci CMLS.

[19] Uluçkan Ö, Segaliny A, Botter S, Santiago JM, Mutsaers AJ (2015). Preclinical mouse models of osteosarcoma. BoneKEy Rep.

[20] Lee RJ, Arshi A, Schwartz HC, Christensen RE (2015). Characteristics and prognostic factors of osteosarcoma of the jaws: a retrospective cohort study. JAMA Otolaryngol Head Neck Surg.

[21] Ory B, Heymann M-F, Kamijo A, Gouin F, Heymann D, Redini F (2005). Zoledronic acid suppresses lung metastases and prolongs overall survival of osteosarcoma-bearing mice. Cancer.

[22] Kilkenny C, Browne WJ, Cuthill IC, Emerson M, Altman DG (2010). Improving bioscience research reporting: the ARRIVE guidelines for reporting animal research. PLoS Biol.

[23] Janeway KA, Walkley CR (2010). Modeling human osteosarcoma in the mouse: from bedside to bench. Bone.

[24] Walia MK, Castillo-Tandazo W, Mutsaers AJ, Martin TJ, Walkley CR (2017). Murine models of osteosarcoma: a piece of the translational puzzle. J Cell Biochem.

[25] Blattmann C, Thiemann M, Stenzinger A, Roth EK, Dittmar A, Witt H (2015). Establishment of a patient-derived orthotopic osteosarcoma mouse model. J Transl Med.

[26] Mutsaers AJ, Ng AJM, Baker EK, Russell MR, Chalk AM, Wall M (2013). Modeling distinct osteosarcoma subtypes in vivo using Cre:lox and lineage-restricted transgenic shRNA. Bone.

[27] Jones KB (2011). Osteosarcomagenesis: modeling cancer initiation in the mouse. Sarcoma.

[28] Lai Y, Wei X, Lin S, Qin L, Cheng L, Li P (2017). Current status and perspectives of patient-derived xenograft models in cancer research. J Hematol OncolJ Hematol Oncol.

[29] Stebbing J, Paz K, Schwartz GK, Wexler LH, Maki R, Pollock RE (2014). Patient-derived xenografts for individualized care in advanced sarcoma. Cancer.

[30] Houghton PJ, Morton CL, Tucker C, Payne D, Favours E, Cole C (2007). The pediatric preclinical testing program: description of models and early testing results. Pediatr Blood Cancer.

[31] Bousquet G, Janin A (2016). Patient-derived xenograft: an adjuvant technology for the treatment of metastatic disease. Pathobiol J Immunopathol Mol Cell Biol.

[32] Joliat MJ, Umeda S, Lyons BL, Lynes MA, Shultz LD (2002). Establishment and characterization of a new osteogenic cell line (MOS-J) from a spontaneous C57BL/6J mouse osteosarcoma. Vivo Athens Greece.

[33] Dass CR, Ek ETH, Choong PFM (2007). Human xenograft osteosarcoma models with spontaneous metastasis in mice: clinical relevance and applicability for drug testing. J Cancer Res Clin Oncol.

[34] Jawad SN, Abdullah BH (2010). Proliferative, apoptotic and angiogenic potentials in jaws and long bones osteosarcomas: a comparative immunohistochemical study. J Oral Pathol Med Off Publ Int Assoc Oral Pathol Am Acad Oral Pathol.

[35] Chen W, Feng H, Li H (2008). Expression and significance of hypoxemia-inducible factor-1alpha in osteosarcoma of the jaws. Oral Surg Oral Med Oral Pathol Oral Radiol Endod.

