# Supplementary material for: Jaw osteosarcoma models in mice: first description
Source: J Transl Med. 2019 Feb 27;17:56. doi: 10.1186/s12967-019-1807-5 (PMC6391788; doi:10.1186/s12967-019-1807-5)

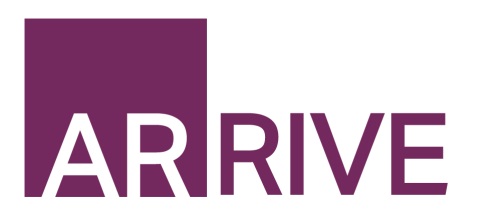


The ARRIVE Guidelines Checklist

Animal Research: Reporting In Vivo Experiments

Carol Kilkenny^1^, William J Browne^2^, Innes C Cuthill^3^, Michael Emerson^4^ and Douglas G Altman^5^

*^1^The National Centre for the Replacement, Refinement and Reduction of Animals in Research, London, UK, ^2^School of Veterinary Science, University of Bristol, Bristol, UK, ^3^School of Biological Sciences, University of Bristol, Bristol, UK, ^4^National Heart and Lung Institute, Imperial College London, UK, ^5^Centre for Statistics in Medicine, University of Oxford, Oxford, UK.*

|  | ITEM | RECOMMENDATION | | Section/ Paragraph |
| --- | --- | --- | --- | --- |
| 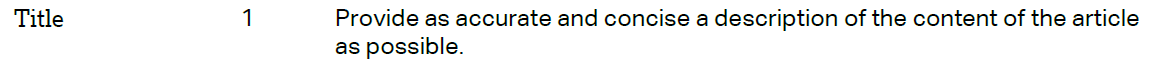 | | | Title | |
| 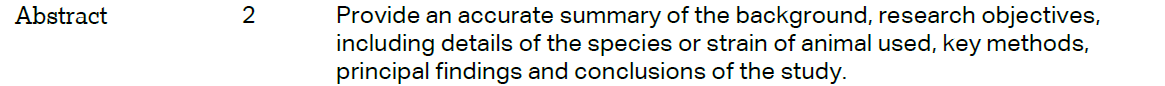 | | | Abstract | |
| INTRODUCTION | | |  | |
| 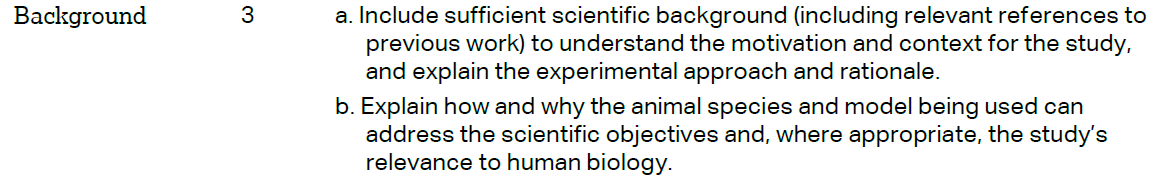 | | | Paragraphs  1-3  Paragraphs  2-3 | |
| 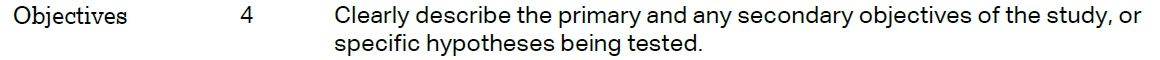 | | | Paragraph 3 | |
| METHODS | | |  | |
| 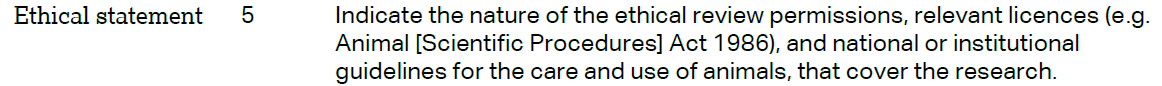 | | | Paragraph 1 | |
| 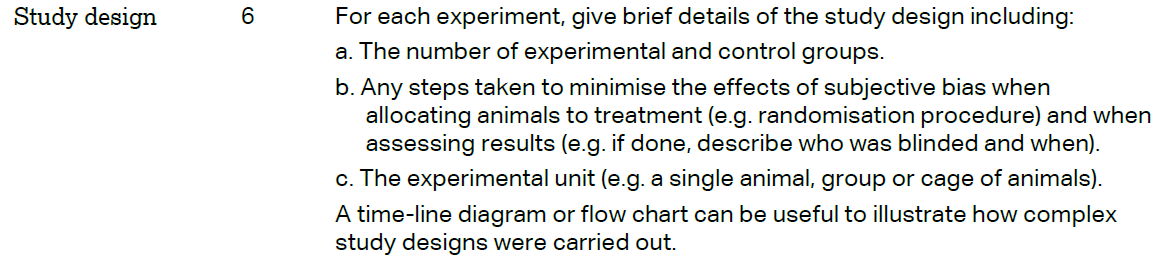 | | | Paragraphs 2, 3, 4 + Fig. 5  Not appplicable  Paragraph 1 | |
| 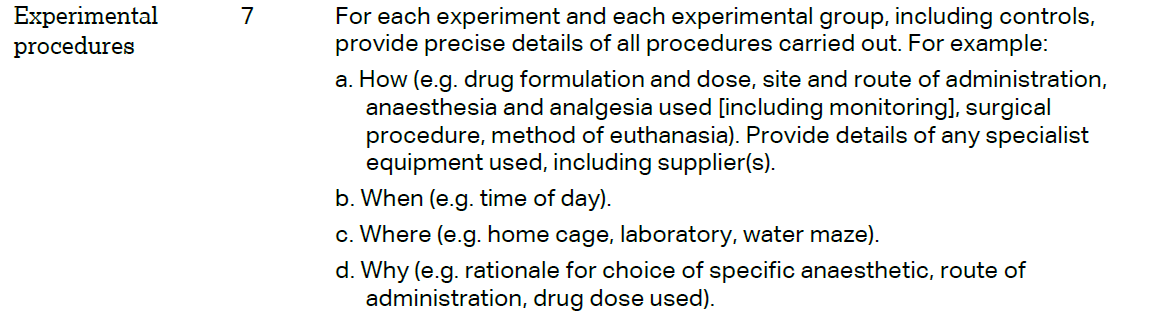 | | | Paragraphs  1-7 | |
| 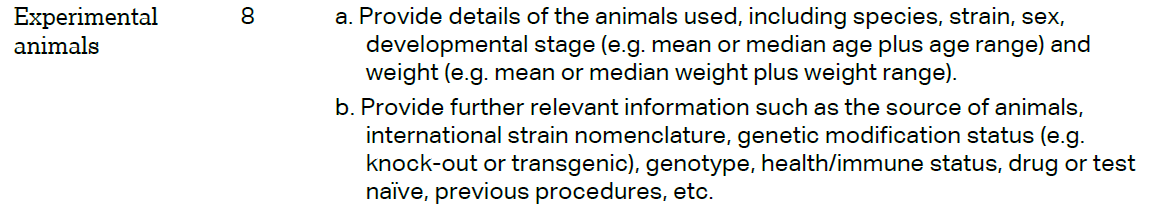 | | | Paragraphs  1-4 | |

The ARRIVE guidelines. Originally published in *PLoS Biology*, June 2010^1^

| 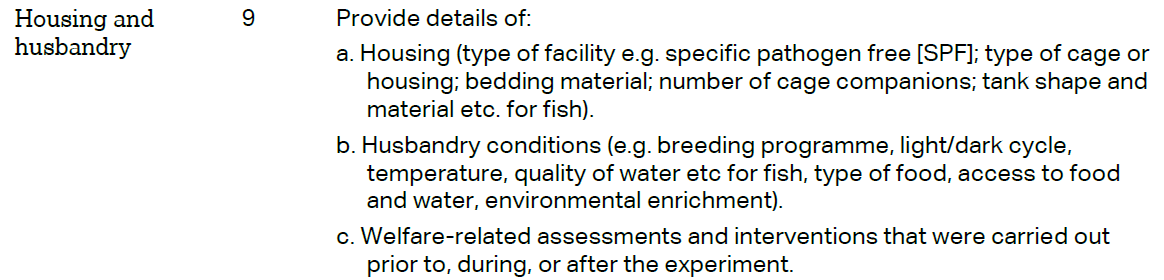 | Paragraph 1 |
| --- | --- |
| 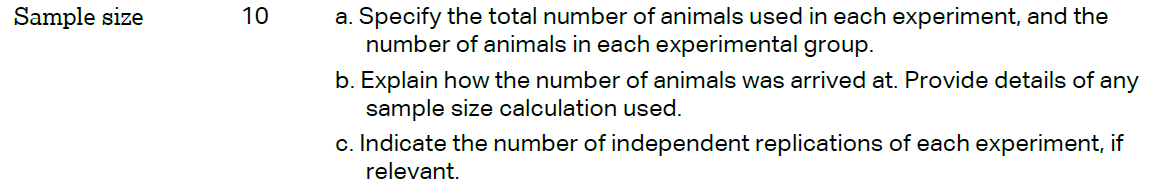 | Paragraphs 1-4 + Fig. 5  + Additional file 2 (Table S1 and figure S1) |
| 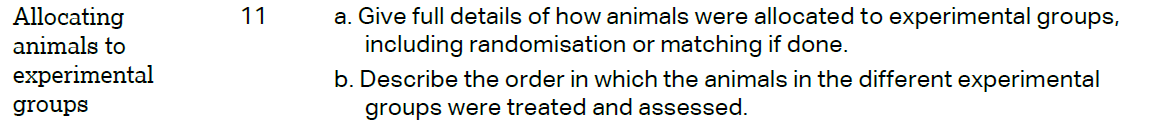 | Not applicable |
| 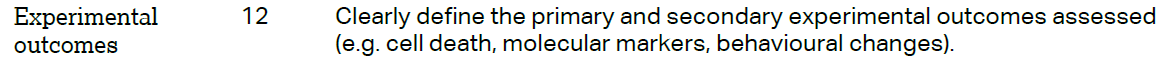 | Paragraphs 3-7 |
| 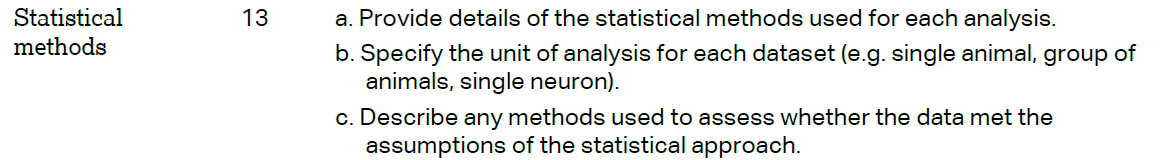 | Paragraph 8 |
| RESULTS |  |
| 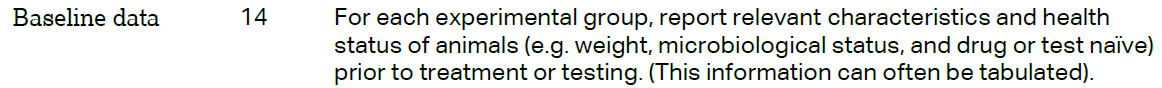 | Not applicable |
| 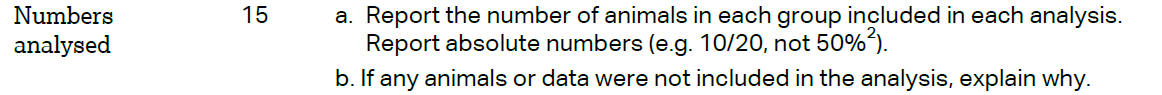 | Table1, figure 5, Additional file 2 (figure S1) |
| 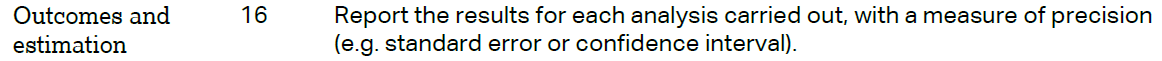 | Paragraphs 1-7  Figures 1-5 |
| 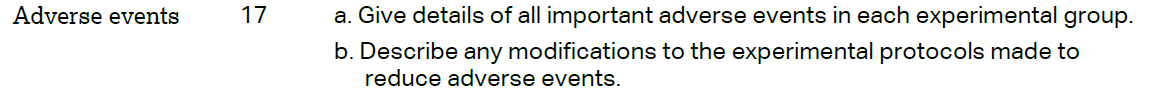 | Not applicable |
| DISCUSSION |  |
| 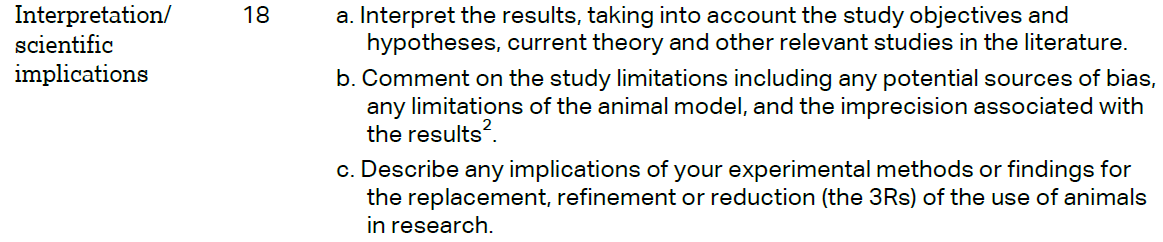 | Paragraphs 3-5  Paragraphs 2-4  Paragraph 4 |
| 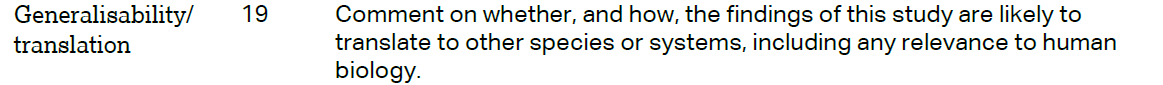 | Paragraphs 1, 2, 4, 6 |
| 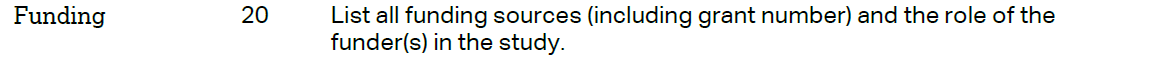 | Funding sources |


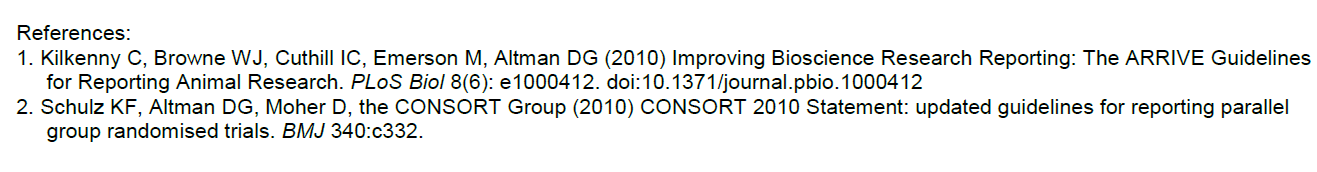

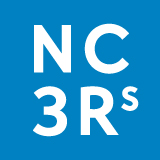

Supplement: Supplementary file 1 — Additional file 1. The arrive guidelines checklist. [file 12967_2019_1807_MOESM1_ESM.docx]
