# Supplementary material for: Jaw osteosarcoma models in mice: first description
Source: J Transl Med. 2019 Feb 27;17:56. doi: 10.1186/s12967-019-1807-5 (PMC6391788; doi:10.1186/s12967-019-1807-5)
Supplement: Supplementary file 2 — Additional file 2: Table S1. Murine and human osteosarcoma cell lines used for the development of syngenic and xenogenic JOS animal models. Figure S1. Mean mandibular tumor volume over time, as a function of tumor cell number injected in the xenogenic HOS1544 (A) or the syngenic MOS-J (B) models in NMRI-nude or C57Bl/6 mice respectively. [file 12967_2019_1807_MOESM2_ESM.docx]

| Origin | Species | Cell lines | Source |
| --- | --- | --- | --- |
| Long bones osteosarcoma | Mouse | MOS-J | Jackson Laboratoy^®^ |
|  |  | POS-1 | Jackson Laboratoy^®^ |
|  |  | K7-M2 | ATCC^®^ CRL-2836™ |
| Long bones osteosarcoma | Human | HOS-1544 | ATCC^®^ CRL-1544™ |
|  |  | HOS-1547 | ATCC^®^ CRL-1547™ |
|  |  | MG-63 | ATCC^®^ CRL-1427™ |
|  |  | SaOS-2 | ATCC^®^ HTB-85™ |

*Table S1. Murine and human osteosarcoma cell lines used for the development of syngenic and xenogenic JOS animal models.*


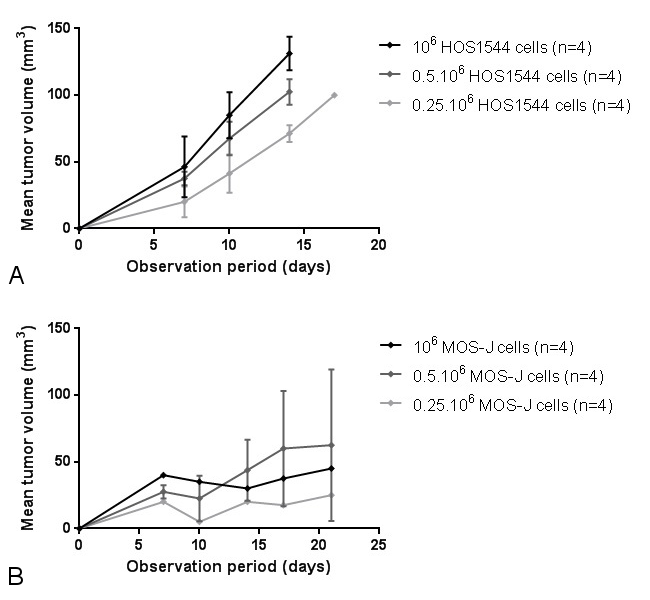


*Figure S1. Mean mandibular tumor volume over time, as a function of tumor cell number injected in the xenogenic HOS1544 (A) or the syngenic MOS-J (B) models in NMRI-nude or C57Bl/6 mice respectively.*
